# Risk selection into supplemental private health insurance in China

**DOI:** 10.1186/s13561-019-0252-8

**Published:** 2019-12-17

**Authors:** Yawen Jiang, Weiyi Ni

**Affiliations:** 1 School of Public Health (Shenzhen), Sun Yat-sen University, Room 215, Mingde Garden #6, 132 East Outer Ring Road, Pan-yu District, Guangzhou, Guangdong China; 2 Medtronic Inc, 9775 Toledo Way, Irvine, California, 92618 USA

**Keywords:** Private, Health insurance, Risk, Selection, Advantageous

## Abstract

**Background:**

Information on risk selection is important for the regulation and development of supplemental private health insurance (PHI). The research on risk selection into supplemental PHI has been documented in several developed countries where the regulation of the PHI markets was relatively mature. However, evidence on this important aspect of the supplemental PHI market in China is still absent in the literature. The private insurers in China were not prohibited from discrimination against pre-existing conditions and did not guarantee ongoing enrolment. Therefore, the direction and degree of risk selection could not be inferred using the evidence from the other countries. To provide evidence on risk selection into supplemental PHI in China, we conducted a cross-sectional analysis using data from the 2015 wave of China Health and Retirement Longitudinal Study (CHARLS).

**Results:**

Using probit models, we found that individuals having better self-reported general health were more likely to enrol in PHI in China, suggesting advantageous selection. This result was confirmed by an alternative analysis using an instrumental variable. We also adjusted the realized occurrence of hospitalization by excluding potential moral hazard effect and showed that the adjusted hospitalization risk was negatively associated with PHI enrolment, which also indicated advantageous selection.

**Conclusions:**

The findings suggested potential over-insurance of healthier individuals or under-insurance of less healthy individuals. The regulation of the PHI market in China should aim to address the inefficiency. The current study could also contribute to the information base for policymakers in countries where the PHI markets similarly lack strong regulation.

## Introduction

China experienced fast economic growth since late 1970s. However, the increase of health insurance coverage was not observed alongside with the growth of economy in China [1, 2]. In fact, the population health insurance coverage rate dropped from 73% to 50% during 1993 to 2003 [1]. To reverse this, the Chinese government deployed programs towards universal coverage since 2003 that collectively led to a basic health insurance coverage rate of 95% by 2011 [2–4]. Despite this achievement, the high out-of-pocket (OOP) rate associated with public programs that ranges from 40% to 70% remains a financial challenge to the covered population [3, 5–7]. The reimbursement rate of public insurance is not expected to increase substantially in the near future because the public medical insurance fund in China is already on the verge of a deficit [4, 8]. To avoid the coverage gap, some people purchase private health insurance (PHI) in addition to their public health insurance [2, 7]. Indeed, the Chinese government is engaging private health insurers to provide supplemental coverage of expensive healthcare services [3, 4]. This is reflected by the fact that the current PHI plans dominantly target at critical illnesses that incur high inpatient costs [2, 6, 9].

A mere 3% of the Chinese population is covered by PHI [7]. Although the total PHI premium in China is surging at 30% annually [5, 8], the PHI products still lack important features of supplemental health insurance. For example, PHIs in China usually do not reimburse based on actual expenditure. Instead, they pay a lump sum for a narrow set of conditions and have no commitment to ongoing enrolment [5, 9]. Such policies account for over 70% of the market [5]. In addition, regulation of price discrimination against individuals with unfavourable health conditions in the PHI market is still absent. At the initial enrolment, insurers may require a physical examination report from enrolees. However, the more popular approach is to ask enrolees to disclose any pre-existing conditions [10]. An observation period after enrolment, mostly 90 days, is used to verify if enrolees have critical illnesses at the time of enrolment. The list of critical illness varies across insurers and plans. Pre-existing conditions will become exceptions in the individuals’ plans. Furthermore, renewal of insurance is extremely unlikely if the benefit of a high-cost hospitalization due to a critical illness had been claimed [5]. Hence, the PHI market in China is still premature and should be improved in numerous attributes [4, 5]. To that end, it is important to understand whether the voluntary enrolment into supplemental PHI is driven by adverse or advantageous selection [5, 9]. However, there is currently a dearth of evidence on risk selection into supplemental PHI in China. Such evidence can help regulators and policymakers to establish an efficient insurance market. Recent government efforts to promote PHI as supplements to basic public health insurance including offering tax breaks for purchasing PHI reiterates the evidence need on risk selection [11]. As such, the current study aims to examine whether PHI in China is associated with adverse selection or advantageous selection.

The classic Rothschild-Stiglitz (R-S) [12] model of insurance markets predicts adverse selection at the competitive equilibrium when a consumer’s risk type is private information. Indeed, the literature of adverse selection in the basic health insurance markets is relatively saturated [13–15]. However, studies on risk selection in the supplemental PHI markets are relatively sparse. In addition, current evidence in this field arguably supports advantageous selection or lack of risk selection more than adverse selection.

Wolfe and Goddeeris [16] found that those with better self-reported health were more likely to purchase Medigap, a type of policy sold by private insurers to supplement basic Medicare plans in the US. Similarly, Fang et al. [17] showed that Medicare beneficiaries who were covered by Medigap tended to be healthier than those without Medigap. On top of that, they further identified income, education, financial planning horizon, and cognitive function including financial literacy as sources of advantageous selection. Extending the work by Fang et al., Keane and Stavrunova [18] found advantageous selection into Medigap when not controlling any variables but found adverse selection which was consistent with the R-S model once race and marital status were controlled in addition to the variables that were controlled in the analysis by Fang et al. Advantageous selection has also been documented outside of the US. Buchmueller et al. [19] evidenced advantageous selection in the Australian supplemental PHI market when testing the correlation between self-reported health and PHI enrolment as well as the correlation between realized hospital expenditure and PHI enrolment.

Cutler et al. [20] attributed the advantageous selection into Medigap to the hypothesis that insurance demand in a market was driven more by risk tolerance heterogeneity than by health risk heterogeneity. To test this, he showed that proxies for risk aversion were positively correlated with owning Medigap policies but negatively correlated with ex post health risk. The theory by Culter et al. was echoed by evidence in the German supplemental PHI market [21], where it has been shown risk averse attitude was positively associated with purchasing PHI.

However, lack of risk selection into supplemental PHI has also been found. Such findings were recently documented for several European countries and Israel [22, 23]. In both studies, self-reported health was not significantly associated with PHI enrolment. As such, risk selection into PHI varies across countries. A possible reason of the variation is that basic insurance provided by governments that aim to optimize social efficiency can address adverse selection to a certain extent. Therefore, PHI markets have fewer high-risk consumers than the basic insurance markets [15].

The heterogeneity in the direction of risk selection highlights the importance of examining the PHI market by country to provide specific evidence on potential social inefficiency and policy implication for the regulation. So far, the English literature of risk selection into supplemental insurance exclusively focused on developed countries. The current study provides initial evidence on risk selection in the PHI market in a developing country. In addition, all the markets investigated previously prohibited insurers from price discrimination based on conditions either partially or entirely. Thus, our study also contributes to the literature by documenting risk selection into PHI in a market lacking similar regulation.

### Theoretical background

The R-S model of insurance purchase applies most appropriately to a fully competitive market with asymmetric information [12]. In this scenario, the insured keep their health status as private information such that the insurers cannot practice medical underwriting. Hence, the sicker individuals would buy more insurance. This framework does not directly apply to China in that PHI is supplemental to public health insurance and that the insurers are allowed to price-set using the health information available. Suppose there is a high-risk type individual *H* whose probability of incurring a healthcare expenditure *E* not covered by the public health insurance is p^H^ and a low-risk type individual *L* whose corresponding probability is p^L^ such that we know 0 ≤ p^L^ < p^H^ ≤ 1. Assuming the insurers can successfully screen bad risk, then we have Pr(L) ≥ Pr(H) in the R-S framework in which there is no risk preference heterogeneity where Pr is the probability of possessing PHI for the corresponding risk type.

More, the R-S model does not allow correlation between the risk averse parameter τ and the probability p ∈[0, 1] of incurring a healthcare expenditure *E*. To illustrate how such correlation impacts risk selection, let the expected utilities of purchasing and not purchasing PHI for an individual with wealth *W* and a premium *m* of purchasing PHI be respectively

$$ {\displaystyle \begin{array}{c}{\mathrm{V}}_{\mathrm{PHI}}\left(p,\tau \right)=u\left(W-m\right)+e\\ {}{\mathrm{V}}_{\mathrm{NoPHI}}\left(p,\tau \right)= pu\left(W-E\right)+\left(1-p\right)u(W)\end{array}} $$

where *e* is the fixed non-monetary costs of obtaining PHI and follows a logistic distribution independent of *p* and *τ*, and *u* is the constant relative risk aversion utility function that takes the form $u(y)=\frac{Y^{1-\tau }}{1-\tau }$ [17]. The probability for this individual to buy PHI is then given by

$$ {\Pr}_{\mathrm{PHI}}\left(p,\tau \right)=\frac{\exp \left[{\mathrm{V}}_{\mathrm{PHI}}\left(p,\tau \right)\right]}{\exp \left[{\mathrm{V}}_{\mathrm{PHI}}\left(p,\tau \right)\right]+\exp \left[{\mathrm{V}}_{\mathrm{NoPHI}}\left(p,\tau \right)\right]} $$

which is the form of logistic probability. Since *τ* measures risk aversion, Pr_PHI_(*p*, *τ*) is increasing in *τ* [17]. Fang et al. has shown that, with the additional assumption of negative correlation between *p* and *τ*, Pr_PHI_(*p*, *τ*) may be decreasing in *p* if corr(p, τ) is unaccounted for in analysis [17], leading to advantageous selection.

Following the theories of deviations from the classic R-S model described above, we hypothesize that there is advantageous selection in the supplemental PHI market in China when both the price-setting variables (e.g. demographic information and physical conditions) and the risk tolerance proxies are not adjusted in analysis. Without knowing the correlation between *p* and *τ* a priori, we further hypothesize that there is adverse selection if the price-setting factors are adjusted with the assumption of no correlation between *p* and *τ*. If there is still advantageous selection, then *p* and *τ* are indeed negatively correlated. If so, we hypothesize that adverse selection can be retrieved as predicted by the R-S model if risk tolerance proxies are adjusted.

## Methods

### Data

We conducted a cross-sectional analysis using data from the 2015 wave of China Health and Retirement Longitudinal Study (CHARLS). CHARLS is a longitudinal aging survey of 45 years and older people and their spouses in China with biennial follow-up [7]. It was designed to allow nationally representative estimates using a multistage probability sampling [24, 25]. The first wave of survey in 2011 included 17,708 respondents [24]. The 2015 wave data included 20,284 respondents with positive cross-sectional weights. This survey collected information on 1) socioeconomic information including age, sex, education, residence (rural or urban), assets, income, working status, commercial pension, and health insurance status; 2) health information including self-reported general health, thirteen physician-diagnosed chronic conditions, memory problem, past-year total and OOP inpatient costs, and past-month total and OOP outpatient costs; and 3) behavioural questions including smoking status and alcohol ingestion frequency. Additional information on CHARLS can be found elsewhere [24, 26]. We analysed the subsample that had public health insurance coverage to investigate the properties of PHI as supplemental insurance.

### Empirical methods

To test adverse or advantageous selection into PHI, we regressed PHI enrolment on self-reported general health, which is a proxy for health risk. The studies in the literature described in the literature review section were mixed in terms of the proxy for health risk that was used. Specifically, some studies used self-reported health and some used realized healthcare expenditure or medical occurrence. Whereas realized utilization has the benefit of better reflecting the true health risk, this information is unknown to the enrolees at the time of purchasing PHI. On the contrast, self-reported heath measures how the individuals perceive their own health risk. Therefore, testing risk selection based on self-reported health reflects the spontaneous behaviour as a result of perceived health and corresponding risk tolerance. The self-reported general health had five categories (1 excellent, 2 very good, 3 good, 4 fair, 5 poor). We created a general health indicator (GHI) variable for fair or better health because the median category was fair. Three probit models of PHI on the GHI variable using different specifications were conducted. First, we estimated a univariate model:

$$ {\varPhi}^{-1}\left[\mathit{\Pr}\left({PHI}_i=1|\  GH{I}_i\right)\right]={\alpha}_0+{\alpha}_1 GH{I}_i\left(\ 1\ \right) $$

where *PHI*_*i*_ is an indicator for PHI coverage and *Φ*^−1^ is the probit link function. Second, we controlled age, sex, rural or urban residence, and chronic illness. These variables represent information that insurers might use to price insurance or to select enrolees:

$$ {\varPhi}^{-1}\left[\mathit{\Pr}\left({PHI}_i=1|\  GH{I}_i,{X}_i\right)\right]={\alpha}_0+{\alpha}_1 GH{I}_i+{X}_i^{\prime }{\alpha}_2\left(\ 2\ \right) $$

where *X*_*i*_ is the vector of demographic variables and chronic conditions. Finally, we added variables that were not observed or could not be verified at the time of enrolment but represented potential sources of advantageous selection [17, 18, 20]. These variables included total wealth (in ¥1000), annual income (in ¥1000), smoking, alcohol ingestion frequency (daily or more often), having a commercial pension, and education level (high school or above). The equation form is:

$$ {\varPhi}^{-1}\left[\mathit{\Pr}\left({PHI}_i=1|\  GH{I}_i,{X}_{i,}\ {\mathrm{Y}}_i\right)\right]={\alpha}_0+{\alpha}_1 GH{I}_i+{X}_i^{\prime }{\alpha}_2+{Y}_i^{\prime }{\alpha}_3\left(\ 3\ \right) $$

where Pr() is the probability of the event in the parentheses, *Y*_*i*_ represents the vector of the aforementioned additional variables. In all three equations, *α*_1_ represents risk selection. In equation [2], *α*_1_ represents risk selection conditional on price-setting factors. In equation [3], *α*_1_ represents risk selection conditional on possible sources of advantageous selection that would be relevant if regressions of [2] and [3] suggested advantageous selection. In the results section, we present not only the coefficient estimates but also the marginal effect of GHI at the means on the probability of enrolling in PHI because the latter gives practical implications of the results. In the meantime, we still rely on the coefficient estimates for statistical inference because testing of marginal effects involves estimates of all coefficients instead of only the coefficient of interest and depends on at what values the variables are evaluated [27].

However, residual confounding that correlates with both self-reported health and enrolment into PHI might still exist even though we controlled for socioeconomic, medical and behavioural variables. As a narrative example, pessimism may correlate with not only self-rating of health but also risk attitude and PHI enrolment. To address this layer of endogeneity, we used an instrumental variable (IV) approach. In addition, *α*_1_ in equations [1]– [3] may represent the mix of ex ante risk selection and potential moral hazard if the latter existed. This is sometimes referred to as reverse causality in literature. To disentangle the risk selection effect from potential moral hazard, we adopted a second additional identification strategy for the specification in equation 3 using adjusted realized hospitalization as the proxy for health risk.

In the first additional strategy, an IV represents exogenous randomness that correlates with GHI but not *ϵ*_*i*_ [28]. To that end, we exploited the order effect from the design of the CHARLS survey. Order effect refers to the phenomenon that the order of presenting the general health question and specific health questions can affect the answer to the general health question [29]. In the CHARLS survey, about 50% of the respondents were randomized to answer the general health question at the beginning of the health and healthcare section and the rest the other way around. We created an indicator variable for answering the question at the beginning and used it as the IV for GHI. This IV is potentially ideal because of randomization. The reason that the answer to the general health question can be affected by the order is that a long list of health-related items presented before answering the general health question may help the respondents to contextualize their health problems or lack thereof, reminding them of how sick or healthy they are. More formally, self-perceived health can be decomposed into:$Self\ {perceived\ health}_i={SPH}_{i\_ pre}+{SPH}_{i\_ post}+{v}_{\mathrm{i}}+{r}_i\left(\ 4\ \right)$ where *SPH*_*i* _ *pre*_ is the part of self-perceived health unaffected by first answering the other health-related items, *SPH*_*i* _ *post*_ is the part that was affected and was null for those randomized to report general health at the beginning, *v*_i_ is an endogenous component that correlated with both self-perception of health and PHI enrolment, and *v*_i_ is the remaining component that was assumed to be random. Naturally, the order of answering the question affects *GHI*_*i*_ through *SPH*_*i* _ *post*_, which is itself a reflection of part of the true underlying health status. The condition of correlation with GHI was tested in the IV regressions. The Staiger-Stock rule of thumb is that the F-statistic of regressing the endogenous variable on the IV in a linear model should be greater than 10 [30]. Therefore, we also conducted a side-track linear regression of GHI on the order of answering the general health question to examine the F-statistic. It should also be noted that both the response variable of interest (PHI enrolment) and the potentially endogenous treatment variable (GHI) were binary outcomes. Studies have shown that the conventional two-stage least squares procedure of implementing IV leads to misspecification of the second stage and is more vulnerable to bias than bivariate probit (BVP) models when both the response variable and the endogenous variable are binary [31–33]. Therefore, we used the BVP approach to implement the IV analysis. The structure of the BVP model takes the following form [28]:

$$ {\displaystyle \begin{array}{c}{PHI}_i=1\left\{{\alpha}_0+{\alpha}_1 GH{I}_i+{X}_i^{\prime }{\alpha}_2+{Y}_i^{\prime }{\alpha}_3+{\epsilon}_{1i}>0\right\},\\ {}{GHI}_i=1\left\{{\beta}_0+{\beta}_1{OI}_i+{\epsilon}_{2i}>0\right\},\\ {}\left(\genfrac{}{}{0pt}{}{\epsilon_{1i}}{\epsilon_{2i}}|\ {X}_i,{Y}_i,O{I}_i\right)\sim BVN\ \left[\left(\genfrac{}{}{0pt}{}{0}{0}\right),\left(\begin{array}{cc}1& \rho \\ {}\rho & 1\end{array}\right)\right],\end{array}}\left(\ 5\ \right) $$

where 1{} is an indicator function taking the value one if the condition in the curly brackets are true and zero otherwise, *OI*_*i*_ is the indicator of answering the general health question at the beginning, and BVN represents bivariate normal distribution. Conditional on *OI*_*i*_ qualifying as an IV, ρ tests the exogeneity of GHI to PHI. This regression was conducted using the maximum likelihood approach.

The second additional identification strategy was replacing GHI with the adjusted risk of hospitalization. We followed the approach used by Fang et al. [17] to analyse risk selection in the Medigap market by using the adjusted risk of hospitalization as the proxy for the underlying health risk. First, the realized occurrence of hospitalization was regressed on the same set of predictors as in equation 3 using a probit model:

$$ {\varPhi}^{-1}\left[\mathit{\Pr}\left({H}_i=1|\  PH{I}_i,{X}_{i,}\ {Y}_i\right)\right]={\gamma}_0+{\gamma}_1 PH{I}_i+{X}_i^{\prime }{\gamma}_2+{Y}_i^{\prime }{\gamma}_3\left(\ 6\ \right) $$

where *H*_*i*_ is an indicator for had any hospitalization in the past year and *γ*_1_ represents potential moral hazard. Then, the adjusted risk of hospitalization is calculated as the predicted probability of hospitalization using the results of equation 6 but excluding the effect of PHI:

$$ {\hat{P}}_i=\varPhi \left({\hat{\gamma}}_0+{X}_i^{\prime }{\hat{\gamma}}_2+{Y}_i^{\prime }{\hat{\gamma}}_3\right)\left(\ 7\ \right) $$

where *Φ* is the standard normal cumulative density function. Finally, the specification in equation 3 was re-analysed using:

$$ {\varPhi}^{-1}\left[\mathit{\Pr}\left({H}_i=1|\  PH{I}_i,{X}_{i,}\ {Y}_i\right)\right]={\delta}_0+{\delta}_1{\hat{P}}_i+{X}_i^{\prime }{\delta}_2+{Y}_i^{\prime }{\delta}_3.\left(\ 8\ \right) $$

Similar to *α*_1_ in equations 3 and 5, *δ*_1_ represents risk selection. However, the interpretation of the direction of the effect of ${\hat{\mathrm{P}}}_{\mathrm{i}}$ is opposite to that of GHI. Since PHI plans in China do not reimburse outpatient visits, the high-risk and low-risk individuals would likely differ in the underlying hospitalization risk if there was risk selection.

All analyses used respondent sampling weights and were conducted using Stata (version 14; Stata Corp, College Station, TX, USA).

## Results

We identified 17,704 respondents who had public health insurance in the 2015 cross-sectional data, 493 of them had PHI. A tabulation incorporating sampling weights showed that 3.2% of those who had public health insurance also had PHI. Table 1 lists descriptive statistics of the analytical sample. Briefly, PHI beneficiaries were significantly younger (mean: 53.8 vs. 60.1 years, *p* < 0.001), less likely to live in rural areas (30.2% vs. 52.5%, p < 0.001), and more likely to be in fair or better health status (90.4% vs 80.3%, p < 0.001). PHI beneficiaries also had significantly higher annual income (mean: ¥20,695 vs. ¥6165, *p* = 0.001).

**Table 1:** Characteristics of individuals having public health insurance with and without PHI in CHARLS

	With PHI (3.2%)^a^	Without PHI (96.8%)^a^	Total	*p*-value
Age (years)	53.8 (0.4)	60.1 (0.2)	59.9 (0.1)	< 0.001
Male (%)	50.4	47.6	47.7	0.462
Rural (%)	30.2	52.5	51.8	< 0.001
Mean number of chronic conditions	1.62 (0.12)	1.77 (0.02)	1.76 (0.02)	0.243
Self-reported health status (%)				< 0.001
Excellent	2.3	1.3	1.3	
Very good	15.9	11.7	11.8	
Good	15.8	12.8	12.9	
Fair	56.5	54.6	54.6	
Poor	9.6	19.7	19.4	
Total wealth (2015 Chinese ¥)	570,057(64,788)	455,544(31,662)	459,265(30,697)	0.112
Income (2015 Chinese ¥)	20,695(4289)	6165(241)	6622(272)	0.001
1-year inpatient costs (2015 Chinese ¥)	2463(115)	1900(858)	1918	0.516
Had any inpatient admission (%)	11.9	13.2	13.1	0.468

Results are presented as mean (standard error) unless otherwise specified

*CHARLS* China Health and Retirement Longitudinal Study, *PHI* private health insurance

^a^The percentages incorporated sampling weights. Therefore, the actual sample sizes in each group were not reported because they did not correspond to the reported percentages

Estimates of coefficients and marginal effects for equations [1]– [3] are presented in Tables 2, 3 and 4, respectively. In the unadjusted equation, GHI was significantly associated with a 0.0252 (*p* < 0.001) higher probability of having PHI. When conditional on potential price setting and consumer selection variables, GHI was significantly associated with a 0.0142 (*p* < 0.05) higher probability of having PHI. In equation [3], only having commercial pension among all the variables of potential sources of advantageous selection was significantly associated with PHI enrolment. Also, GHI was still significantly associated with a 0.0182 (p < 0.05) higher probability of PHI enrolment, which was relatively close to the magnitude in equation [2].

**Table 2:** Estimates of the coefficient and marginal effect from the unadjusted probit regression of supplemental private health insurance enrolment on general health indicator (equation 1)

	Coefficient	Marginal effect
General Health Indicator^a^	0.350^***^ (0.0698)	0.0252^***^ (0.00572)
N	19,275	

Standard errors in parentheses

^*^ *p* < 0.05, ^**^ *p* < 0.01, ^***^ *p* < 0.001

^a^The general health indicator was 1 if self-reported health was fair or better than fair and was 0 if self-reported health was poor. Zero was the baseline category in regression

**Table 3:** Estimates of the coefficients and marginal effects from the probit regression of supplemental private health insurance enrolment on general health indicator adjusting for demographic variables and physical conditions (equation 2)

	Coefficient	Marginal effect
General Health Indicator^a^	0.214^*^ (0.0926)	0.0142^*^ (0.00648)
Live in rural area	−0.357^***^ (0.0772)	− 0.0237^***^ (0.00625)
Age	−0.0301^***^ (0.00448)	− 0.00199^***^ (0.000359)
Male	0.0730 (0.0837)	0.00484 (0.00549)
Ever had condition		
High blood pressure	0.0406 (0.113)	0.00269 (0.00756)
Diabetes	−0.168 (0.140)	−0.0112 (0.00938)
Cancer	0.0485 (0.261)	0.00322 (0.0173)
Lung disease	−0.335^**^ (0.127)	−0.0222^*^ (0.00897)
Heart problem	0.0960 (0.105)	0.00637 (0.00684)
Stroke	−0.166 (0.219)	−0.0110 (0.0147)
Psychiatric problem	0.668 (0.390)	0.0443 (0.0268)
Arthritis	−0.0756 (0.0878)	−0.00501 (0.00588)
Dyslipidaemia	0.0404 (0.106)	0.00268 (0.00698)
Liver disease	0.245 (0.188)	0.0163 (0.0127)
Kidney disease	0.354 (0.246)	0.0235 (0.0171)
Stomach/digestive disease	−0.144 (0.0968)	−0.00954 (0.00656)
Asthma	0.174 (0.176)	0.0115 (0.0117)
Memory problem	0.383 (0.304)	0.0254 (0.0204)
N	12,929	

Standard errors in parentheses

^*^ *p* < 0.05, ^**^ *p* < 0.01, ^***^ *p* < 0.001

^a^The general health indicator was 1 if self-reported health was fair or better than fair and was 0 if self-reported health was poor. Zero was the baseline category in regression

**Table 4:** Estimates of the coefficients and marginal effects from the probit regression of supplemental private health insurance enrolment on general health indicator adjusting for demographic variables, physical conditions, and risk tolerance proxies (equation 3)

	Coefficient	Marginal effect
General Health Indicator^a^	0.281^*^ (0.116)	0.0182^*^ (0.00809)
Live in rural area	−0.319^**^ (0.0983)	− 0.0207^**^ (0.00755)
Age	−0.0222^***^ (0.00568)	−0.00144^***^ (0.000406)
Male	−0.0604 (0.133)	−0.00392 (0.00872)
Ever had condition		
High blood pressure	0.0767 (0.128)	0.00498 (0.00847)
Diabetes	−0.146 (0.171)	− 0.00950 (0.0111)
Cancer	0.0896 (0.322)	0.00582 (0.0208)
Lung disease	−0.323^*^ (0.155)	−0.0210^*^ (0.0107)
Heart problem	0.112 (0.128)	0.00727 (0.00809)
Stroke	−0.823^*^ (0.413)	−0.0534 (0.0278)
Psychiatric problem	−0.499 (0.380)	− 0.0324 (0.0251)
Arthritis	−0.0969 (0.112)	− 0.00629 (0.00736)
Dyslipidaemia	0.00348 (0.128)	0.000226 (0.00828)
Liver disease	−0.0548 (0.173)	− 0.00356 (0.0113)
Kidney disease	0.507 (0.270)	0.0329 (0.0189)
Stomach/digestive disease	−0.0487 (0.121)	−0.00316 (0.00789)
Asthma	0.0994 (0.227)	0.00646 (0.0147)
gMemory problem	0.586 (0.345)	0.0381 (0.0231)
Household total wealth (in 1000 Chinese ¥)	0.00000417 (0.00000866)	0.000000271 (0.000000559)
Annual personal income (in 1000 Chinese ¥)	0.00424 (0.00280)	0.000275 (0.000190)
Smoke now	0.0487 (0.123)	0.00316 (0.00798)
Drink alcohol daily or more often	−0.115 (0.129)	−0.00748 (0.00855)
Have commercial pension	1.046^***^ (0.282)	0.0680^***^ (0.0183)
Education high school or above	0.155 (0.135)	0.0100 (0.00865)
N	9403	

Standard errors in parentheses

^*^ *p* < 0.05, ^**^ *p* < 0.01, ^***^ *p* < 0.001

^a^The general health indicator was 1 if self-reported health was fair or better than fair and was 0 if self-reported health was poor. Zero was the baseline category in regression

Table 5 displays the coefficient and marginal effect estimates of IV regression in equations 5 using the BVP approach. Although the regression was estimated using maximum likelihood, we followed the convention of IV analysis to term the second equation in equations 5 as the first-stage regression and the first equation in equations 5 as the second-stage regression. In the first-stage regression, the OI variable significantly and negatively predicted the response to the general health question, supporting its validity as an IV. The F-statistic of the side-track first-stage linear regression was 109.42, which was substantially greater than the Staiger-Stock rule of thumb for non-weak IV. In the second stage regression, GHI was significantly associated with a 0.244 (*p* < 0.001) higher probability of having PHI, confirming the results of advantageous selection. Furthermore, *ρ* was statistically significant, suggesting endogeneity of GHI to PHI.

**Table 5:** Coefficients and marginal effects at the means of the bivariate probit regression of supplemental private health insurance enrolment on general health indicator using the order of answering the general health question as an instrumental variable

	Coefficient	Marginal effect at the means
*First-stage regression*		
Answering the general health question at the beginning of health section	−0.258^***^ (0.0426)	− 0.0690^***^ (0.0113)
*Second-stage regression*		
General Health Indicator^a^	1.574^***^ (0.184)	0.244^**^ (0.0751)
Live in rural area	−0.213^**^ (0.0687)	− 0.0330^**^ (0.0109)
Age	− 0.0153^***^ (0.00465)	− 0.00238^***^ (0.000598)
Male	−0.0276 (0.0910)	− 0.00427 (0.0139)
Ever had condition		
High blood pressure	0.0393 (0.0851)	0.00609 (0.0131)
Diabetes	− 0.0990 (0.116)	−0.0154 (0.0181)
Cancer	0.0788 (0.236)	0.0122 (0.0365)
Lung disease	−0.228^*^ (0.114)	− 0.0353^*^ (0.0171)
Heart problem	0.0713 (0.0919)	0.0111 (0.0136)
Stroke	−0.542 (0.295)	−0.0840 (0.0449)
Psychiatric problem	−0.382 (0.260)	− 0.0592 (0.0402)
Arthritis	−0.0755 (0.0764)	− 0.0117 (0.0116)
Dyslipidaemia	0.0173 (0.0883)	0.00268 (0.0137)
Liver disease	−0.0117 (0.121)	−0.00182 (0.0187)
Kidney disease	0.353 (0.191)	0.0547 (0.0285)
Stomach/digestive disease	−0.0347 (0.0837)	− 0.00538 (0.0130)
Asthma	0.0495 (0.167)	0.00767 (0.0255)
Memory problem	0.408 (0.236)	0.0632 (0.0380)
Household total wealth (in thousand Chinese ¥)	0.00000366 (0.00000679)	0.000000568 (0.00000105)
Annual personal income (in thousand Chinese ¥)	0.00286 (0.00215)	0.000443 (0.000315)
Smoke now	0.0395 (0.0835)	0.00612 (0.0130)
Drink alcohol daily or more often	−0.0750 (0.0866)	−0.0116 (0.0138)
Have commercial pension	0.783^**^ (0.240)	0.121^***^ (0.0331)
Education high school or above	0.117 (0.0902)	0.0182 (0.0140)
**ρ**	−1.243^***^ (0.328)	
N	9403	

Standard errors in parentheses

^*^ *p* < 0.05, ^**^ *p* < 0.01, ^***^ *p* < 0.001

^a^The general health indicator was 1 if self-reported health was fair or better than fair and was 0 if self-reported health was poor. Zero was the baseline category in regression

Results of the regression using equation 8 are listed in Table 6. The adjusted risk of hospitalization was significantly and negatively associated with having PHI. This means individuals at higher risks of hospitalization were less likely to purchase PHI. The marginal effect of the adjusted risk of hospitalization was that the probability of PHI enrolment was reduced by 0.108 (*p* < 0.05). This result also confirmed advantageous selection.

**Table 6:** Coefficients and marginal effects at the means of the probit regression of supplemental private health insurance enrolment on the adjusted risk of hospitalization

	Coefficient	Marginal effect at the means
Adjusted risk of hospitalization	−1.668^*^ (0.808)	−0.108 (0.0563)
Live in rural area	−0.338^***^ (0.100)	−0.0219^**^ (0.00772)
Age	−0.0191^***^ (0.00544)	−0.00124^***^ (0.000373)
Male	−0.00874 (0.131)	−0.000567 (0.00851)
Ever had condition		
High blood pressure	0.0883 (0.130)	0.00572 (0.00861)
Diabetes	−0.0383 (0.171)	−0.00248 (0.0111)
Cancer	0.233 (0.324)	0.0151 (0.0210)
Lung disease	−0.224 (0.160)	−0.0145 (0.0106)
Heart problem	0.218 (0.122)	0.0141 (0.00772)
Stroke	−0.781 (0.404)	−0.0506 (0.0271)
Psychiatric problem	−0.518 (0.380)	−0.0336 (0.0252)
Arthritis	−0.108 (0.112)	−0.00698 (0.00731)
Dyslipidaemia	0.0710 (0.128)	0.00460 (0.00829)
Liver disease	0.00233 (0.171)	0.000151 (0.0111)
Kidney disease	0.561^*^ (0.282)	0.0364 (0.0198)
Stomach/digestive disease	−0.0249 (0.119)	−0.00161 (0.00770)
Asthma	0.123 (0.222)	0.00800 (0.0144)
Memory problem	0.687 (0.362)	0.0446 (0.0244)
Household total wealth (in thousand Chinese ¥)	0.00000234 (0.00000844)	0.000000152 (0.000000545)
Annual personal income (in thousand Chinese ¥)	0.00267 (0.00275)	0.000173 (0.000182)
Smoke now	−0.00990 (0.126)	− 0.000642 (0.00816)
Drink alcohol daily or more often	−0.157 (0.133)	− 0.0102 (0.00888)
Have commercial pension	1.051^***^ (0.282)	0.0682^***^ (0.0184)
Education high school or above	0.153 (0.136)	0.00994 (0.00870)
N	9403	

Standard errors in parentheses

^*^ *p* < 0.05, ^**^ *p* < 0.01, ^***^ *p* < 0.001

## Discussion

To our knowledge, the current study is the first in the literature to document risk selection associated with PHI in China. Using CHARLS data, we found that there was advantageous selection into PHI among those who had public health insurance in China, which echoed our first hypothesis.

The advantageous selection was partially mediated by physical conditions, suggesting potential discrimination or selection of enrolees by insurers. However, there was still advantageous selection after controlling for the demographic variables and physical conditions. Previous studies in the literature were conducted in contexts with regulation against discrimination based on pre-existing conditions. Hence, it was natural to expect that physical conditions would explain advantageous selection in a setting that lacked anti-discrimination legislation. On the contrast of our second hypothesis, our findings suggested that advantageous selection could drive enrolment into PHI even though insurers already screened risk on their side.

In the Fang et al. study [17], advantageous selection was explained by the correlation between risk preference variables and health. Similar risk preference variables were also controlled in the current analysis where available, but controlling these variables still did not fully explain the advantageous selection. Whereas it further nullified our third hypothesis, it also likely suggested that there were still unidentified sources of risk preference. For example, risk preference may depend not only on static variables but also on the dynamic change of socioeconomic conditions. Prospect theory predicts that baseline states and changes in wealth can affect risk tolerance [34]. Future studies should try to investigate sources of advantageous selection in a dynamic setting.

The IV approach aimed to address possible omitted variable bias caused by a series of unobserved factors. The unobserved factors could include individual attitude which may contribute to both self-perceived health and enrolment in PHI. However, the results of the IV approach should be interpreted with caution. Our approach targeted at the endogeneity in self-reported health but not health itself. To a certain extent, this resembled measurement error.

An additional concern of omitted variable bias may be raised by the regional variation in how much the public health insurance schemes address the healthcare need. The underlying rationale is that the OOP rates of healthcare expenditure across regions may affect the uptake of PHI. Although there isn’t an ideal approach to quantify the potential omitted variable bias due to this complexity, we conducted a post hoc analysis to provide indirect evidence on whether the regional variation of public health insurance schemes biased the results. First, we created a proxy of the generosity of public health insurance by calculating the city-level OOP rate of past-year inpatient costs using the subsample who had any hospitalization and only had public health insurance, following which we examined if this variable was significantly associated with having PHI. Indeed, the probability of having PHI was negatively associated with the city-level OOP rate of hospitalization. Specifically, the probability of having PHI was increased by 9.7 percentage points (*p* = 0.008) if the OOP rate was changed from 100% to 0%. This suggests that the regional variation of public insurance schemes did have the potential to cause omitted variable bias. Second, we added the city-level OOP rate variable to the covariate list of equation 3 to observe if the estimate of risk selection as denoted by the effect of the general health indicator had any change. The coefficient and marginal effect estimates remained the same as that in Table 4 (0.28 & 0.018), as did the statistical significance of the estimates. More, the city-level OOP rate variable was statistically insignificant in this regression. Hence, we did not find evidence of omitted variable bias caused by regional variation of public health insurance schemes.

In addition to internal validity, which is mainly threatened by omitted variable bias, another dimension of the validity is construct validity. We created a dichotomous general health indicator to test risk selection. However, the results could be misleading if self-reported health could not be dichotomized. We explored the analysis of equation 3 by regrouping categories 1 and 2 of self-reported health into “excellent or very good” and categories 3 and 4 into “good or fair”. Category 5, which represented “poor”, was used as the reference category. The marginal effect of “excellent or very good” health was 0.023 (*p* = 0.043). The marginal effect of the “good or fair” health was 0.0175 (*p* = 0.033). These results not only confirmed the advantageous selection but also documented that the better the health the stronger the advantageous selection. This study has several limitations. First, the data source lacked accurate ex ante health risk information. An ideal dataset would permit researchers to use the PHI enrolment date as the index date and to collect pre-index health and cost information. Second, we might have not sufficiently adjusted for price-setting factors that insurers used for medical underwriting. For example, the insurers may exercise screening of potential beneficiaries, the results of which are not necessarily reported to or recalled by the respondents. This may result in unaccounted-for omitted variable bias. Third, the variables we were able to use as proxies for risk preferences were not as extensive as other studies. This restricted our understanding of the sources of advantageous selection. Fourth, some variables had substantial missing data issues, which led to reduced sample sizes and might have resulted in underpowered analyses. This could be a potential reason that income was significantly different between those with and without PHI in descriptive statistics but was not significantly associated with PHI enrolment in regressions.

Our findings have important policy implications. First, the existence of either adverse selection or advantageous selections may indicate social inefficiency [23]. In the case of advantageous selection, high-risk individuals may be underinsured [18]. Future policies to regulate the PHI market should take this into consideration. Based on the evidence from the current study, developing anti-discrimination policies can allow more high-risk individuals to purchase PHI, thereby counterbalancing the advantageous selection. In addition, to the extent that health and wealth are usually positively correlated, the current tax deduction incentives to purchase supplemental PHI can be redesigned and leveraged to disproportionately favour the low-income high-risk population. Finally, the evidence from the current study could also inform policymakers in countries where the supplemental PHI markets are similarly premature and the public health insurance addresses only a modest portion of healthcare demand.

## Conclusions

To sum up, we used data from CHARLS to provide evidence on advantageous selection in the Chinese PHI market. The findings were confirmed by various identification strategies including multivariate regressions of PHI enrolment on GHI, IV analysis implemented with BVP, and multivariate regressions of PHI enrolment on adjusted risk of hospitalization. We demonstrated that part of the advantageous selection was not necessarily spontaneous but likely imposed by insurers. This finding was more likely to happen in a context lacking regulation against condition-based discrimination. However, there was remaining advantageous selection that could not be explained by medical conditions and risk preference proxy variables. Based on our findings, we recommend that the PHI market regulation and tax deduction incentives in China should favour high-risk individuals.

## Data Availability

The raw data used in the present study is publicly available at http://charls.pku.edu.cn/en.

## References

[1] Ying XH, Hu TW, Ren J, Chen W, Xu K, Huang JH (2007). Demand for private health insurance in Chinese urban areas. Health Econ.

[2] Liu H, Gao S, Rizzo JA (2011). The expansion of public health insurance and the demand for private health insurance in rural China. China Econ Rev.

[3] Yu H (2015). Universal health insurance coverage for 1.3 billion people: what accounts for China's success?. Health policy.

[4] Ng A, Dyckerhoff CS, Then F. Private health insurance in China: Finding the winning formula: McKinsey & Company; 2012. [Available from: https://www.mckinsey.com/industries/healthcare-systems-and-services/our-insights/private-health-insurance-in-china-ifnding-the-winning-formula].

[5] Luo Y, Bossany W, Wong J, Chen C. Oppurtunities Open Up in Chinese Private Health Insurance Beijig, China: Boston Consulting Group; 2016. [Available from: https://www.bcg.com/publications/2016/insurance-health-care-payers-providers-opportunities-open-up-in-chinese-private-health-insurance.aspx.

[6] Bhattacharjya AS, Sapra PK (2008). Health insurance in China and India: segmented roles for public and private financing. Health Aff.

[7] Zhang C, Lei X, Strauss J, Zhao Y (2017). Health insurance and health care among the mid-aged and older Chinese: evidence from the national baseline survey of CHARLS. Health Econ.

[8] Zhao J, Ng A. The rise of private health insurance in China: Ernst & Young Global Limited; 2016. [Available from: http://www.ey.com/gl/en/industries/financial-services/insurance/ey-the-rise-of-private-health-insurance-in-china.

[9] Preker AS, Zweifel P, Schellekens OP (2009). Global marketplace for private health insurance: strength in numbers.

[10] Chan J, Xin E, Wu D, Qu T, Shen S, Zhao J, et al. While paper on China commerical health insurance: Ernst & Young Global Limited; 2018. [Available from: https://www.ey.com/Publication/vwLUAssets/ey-white-paper-on-china-commercial-health-insurance/$File/ey-white-paper-on-china-commercial-health-insurance.pdf].

[11] China State Administration of Taxation. Notice of the Ministry of Finance, the State Administration of Taxation and the China Insurance Regulatory Commission on Promoting Nationwide Pilot Policies for Individual Income Tax on Commercial Health Insurance. Beijing, China2017.

[12] Rothschild M, Stiglitz J. Equilibrium in competitive insurance markets: An essay on the economics of imperfect information. Uncertainty Econ: Elsevier. 1978:257–80.

[13] van de Ven WP, van Vliet RJ (1995). Consumer information surplus and adverse selection in competitive health insurance markets: an empirical study. J Health Econ.

[14] Neudeck W, Podczeck K (1996). Adverse selection and regulation in health insurance markets. J Health Econ.

[15] Boone J. Basic versus supplementary health insurance: access to care and the role of cost effectiveness. J Health Econ. 2018. 10.1016/j.jhealeco.2018.05.002 29913308

[16] Wolfe JR, Goddeeris JH (1991). Adverse selection, moral hazard, and wealth effects in the Medigap insurance market. J Health Econ.

[17] Fang H, Keane MP, Silverman D (2008). Sources of advantageous selection: evidence from the Medigap insurance market. J Polit Econ.

[18] Keane M, Stavrunova O (2016). Adverse selection, moral hazard and the demand for Medigap insurance. J Econ.

[19] Buchmueller TC, Fiebig DG, Jones G, Savage E (2013). Preference heterogeneity and selection in private health insurance: the case of Australia. J Health Econ.

[20] Cutler DM, Finkelstein A, McGarry K (2008). Preference heterogeneity and insurance markets: explaining a puzzle of insurance. Am Econ Rev.

[21] Schmitz H (2011). Direct evidence of risk aversion as a source of advantageous selection in health insurance. Econ Lett.

[22] Paccagnella O, Rebba V, Weber G (2013). Voluntary private health insurance among the over 50s in Europe. Health Econ.

[23] Shmueli A (2001). The effect of health on acute care supplemental insurance ownership: an empirical analysis. Health Econ.

[24] Zhao Y, Hu Y, Smith JP, Strauss J, Yang G (2012). Cohort profile: the China health and retirement longitudinal study (CHARLS). Int J Epidemiol.

[25] Liu C, Yang C, Zhao Y, Ma Z, Bi J, Liu Y (2016). Associations between long-term exposure to ambient particulate air pollution and type 2 diabetes prevalence, blood glucose and glycosylated hemoglobin levels in China. Environ Int.

[26] Chien S, Lin A, Phillps D, Wilkens J, Lee J. Harmonized CHARLS documentation. In: Research UoSCCfEaS, editor. Los Angeles, CA, USA2017.

[27] Greene William (2009). Discrete Choice Modeling. Palgrave Handbook of Econometrics.

[28] Greene WH (2003). Econometric analysis: Pearson education India.

[29] Lee S, Grant D (2009). The effect of question order on self-rated general health status in a multilingual survey context. Am J Epidemiol.

[30] Staiger DO, Stock JH (1994). Instrumental variables regression with weak instruments.

[31] Bhattacharya J, Goldman D, McCaffrey D (2006). Estimating probit models with self-selected treatments. Stat Med.

[32] Freedman DA, Sekhon JS (2010). Endogeneity in probit response models. Polit Anal.

[33] Chiburis RC, Das J, Lokshin M. A practical comparison of the bivariate probit and linear IV estimators: the World Bank; 2011.

[34] Page L, Savage D, Torgler B, editors. Variation in risk seeking behavior in a natural experiment following a large negative wealth shock induced by a natural disaster. ESAM2012 Conference program; 2012: Econometric Society Australasia.

